# Color stability and surface roughness of novel single-shade universal composite resins exposed to staining solutions: an in vitro study

**DOI:** 10.1007/s44445-025-00035-w

**Published:** 2025-07-15

**Authors:** Malin Janson, Anja Liebermann, Christoph Matthias Schoppmeier

**Affiliations:** 1 Department of Prosthetic Dentistry, University of Cologne, Faculty of Medicine and University Hospital Cologne, Kerpener Str. 32, 50931 Cologne, Germany; 2 Polyclinic for Operative Dentistry and Periodontology, University of Cologne, Faculty of Medicine and University Hospital Cologne, Cologne, Germany

**Keywords:** Single-shade universal composites, Color stability, eLAB system, Staining solutions, Surface roughness

## Abstract

This study investigated the color stability and surface roughness of three novel single-shade universal composites after exposure to common staining solutions. A total of 120 specimens (n = 40 per composite) were fabricated from Transcend Universal Composite (TRA), Ecosite One (ECO), and Clearfil Majesty ES-2 Universal (CLA). Specimens were stored at 37 °C in four different staining solutions (artificial saliva, coffee, red wine, matcha tea). Color changes (ΔE₀₀) were assessed at five time points using the eLAB system, and surface roughness (Sa, Sz) was analyzed via 3D laser scanning microscopy. Statistical analysis was performed using a linear mixed model and post-hoc test (Tukey) (p < 0.05). Composite type, staining solution, and exposure duration significantly affected discoloration (p < 0.001). Red wine induced the most pronounced color change (ECO: ΔE₀₀ = 38.9 ± 1.56), while coffee and matcha tea caused similar discoloration (p = 0.164). TRA showed the greatest surface roughness increase; no correlation with color change was observed. The color stability of single-shade composites is influenced by resin matrix composition and staining agent exposure. Tri-modal or nanohybrid composites showed greater color stability than the micro hybrid composite. Surface roughness did not impact discoloration susceptibility.

## Introduction

The long-term color stability of restorative materials is a critical factor in esthetic reconstructive dentistry, as it significantly impacts both the durability and visual longevity of restorations (Paravina et al. 2015). Among the various restorative materials, resin-based composites must withstand continuous exposure to chromogenic substances found in foods, beverages, and saliva, which can compromise their optical and mechanical integrity over time (Checchi et al. 2024; Catelan et al. 2011; Kochman et al. 2020). Multi-shade composites traditionally achieve esthetic outcomes through stratified layering and customized pigmentation techniques (Chen et al. 2024; Ersöz et al. 2022). While effective, these procedures are time-consuming, technique-sensitive, and require a wide inventory of materials. Single-shade universal composites have been developed to overcome these limitations, offering simplified color selection while maintaining essential aesthetic and functional properties such as strength, durability, and adaptability (Leal et al. 2024). The primary advantage of these materials lies in their dynamic color adjustment. The chameleon effect enables a harmonious integration into the tooth structure by scattering and transmitting light (Altınışık and Özyurt 2023). Optimized resin matrix and filler technology ensures precise adjustment of refractive indices, allowing the material to seamlessly integrate with the optical properties of the natural tooth structure. Various single-shade universal composites already exist, among the available single-shade composites, Clearfil Majesty ES-2 Universal (CLA) is widely used and relies on traditional pigment-based color matching. Newer materials such as Ecosite One (ECO) and Transcend Universal Composite (TRA) employ more advanced technologies. ECO incorporates a hybrid matrix of UDMA, Bis-GMA, and EBPADMA, and features NC-1 (Non-Clustering) technology designed to improve filler dispersion and gloss retention (Sideridou et al. 2008). TRA, also UDMA-based, utilizes Resin Particle Match technology, which aims to fine-tune the optical match between resin and filler for improved visual adaptation. Despite these advances, the long-term esthetic performance of single-shade composites under oral conditions remains insufficiently understood. Chromogenic beverages such as wine, coffee, and tea can lead to discoloration by penetrating the resin matrix and interacting with its surface structure (Najman et al. 2023, Ardu et al. 2009, Elmalawany et al. 2023). Moreover, acidic and alcoholic components contribute to filler–matrix degradation, water sorption, and resin swelling, all of which may accelerate pigment uptake and material breakdown (Catelan et al. 2011; Ardu et al. 2009; Elmalawany et al. 2023). Saliva further facilitates the transport of staining agents and promotes hydrolytic degradation, ultimately resulting in diminished microhardness, marginal deterioration, and secondary discoloration (Ardu et al. 2009; Bétrisey et al. 2018, Erturk-Avunduk et al. 2022). Material composition—including resin hydrophilicity, filler morphology, and surface texture—plays a key role in both esthetic and mechanical resilience (Aktu and Ulusoy 2024; FazlioĞLu et al. 2022). Surface roughness not only affects light reflection and color perception but also contributes to plaque accumulation and biological incompatibility (Rohym et al. 2023).

However, despite increasing clinical adoption, no study to date has systematically evaluated both color stability and surface degradation in current-generation single-shade universal composites under conditions simulating daily dietary exposure. This remains a significant gap in the literature, particularly given the esthetic demands and functional longevity expected of these materials in modern dental practice. Accordingly, this study aimed to evaluate both the color change (ΔE₀₀) and surface roughness (Sa, Sz) of three single-shade universal composites (CLA, ECO, TRA) following 28-day immersion in red wine, coffee, matcha tea, and artificial saliva. The following null hypotheses were proposed: (1) There is no significant difference in color change (ΔE₀₀) between the tested single-shade universal composites (CLA, TRA, ECO) after 28 days of exposure to artificial saliva, coffee, red wine and matcha tea. (2) The surface roughness (Sa, Sz) of the single-shade universal composites remains unchanged after 28 days, regardless of the staining media used.

## Methods

### Sample size calculation

The sample size was calculated using G*Power (Heinrich Heine University Düsseldorf), applying an effect size factor f = 0.4, a significance level of α = 0.05, and a power of 80%, based on a previous study (Chen et al. 2024). A minimum of 10 specimens per group was required, resulting in 40 specimens per composite and a total of 120 specimens.

### Fabrication of study samples

**Fig. 1:**
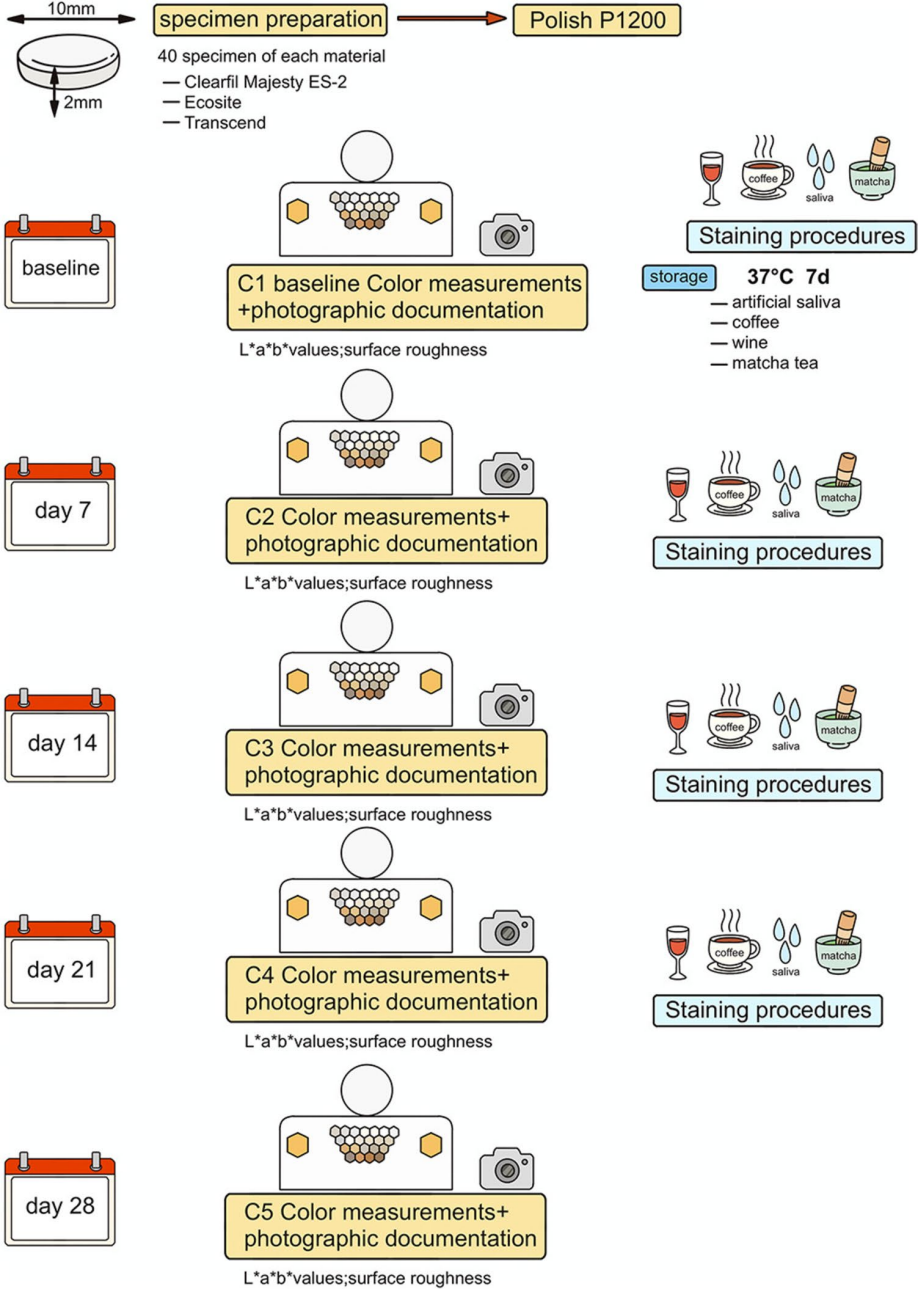
Schematic representation of the study design, including test groups, immersion solutions, and analysis procedures

The study flowchart is presented in Fig. 1. Three different universal single-shade composites were analyzed: CLA (Clearfil Majesty ES-2 Universal, Shade U, Kuraray Noritake, Tokyo, Japan), TRA (Transcend Universal Composite, Shade UB, Ultradent Products, South Jordan, UT, USA), and ECO (Ecosite One, Shade One, DMG, Hamburg, Germany).

The compositions are detailed in Table 1. A total of 120 disc-shaped specimens (n = 40 per composite) with a diameter of 10 mm and a thickness of 2 mm were fabricated. A standardized Teflon mold insert was used to ensure uniform dimensions. The final thickness was verified using a digital caliper (Mitutoyo Corp., Kawasaki, Japan), and specimens that did not meet the minimum thickness were excluded. The composites were placed into the Teflon mold and polymerized for 20 s using an LED light-curing unit (Bluephase Style, 1200 mW/cm^2^, Ivoclar Vivadent, Germany) from both the top and bottom. After removal from the mold, an additional 20-s curing cycle was performed in circular movements according to the DIN EN ISO 4049:2010–03 standard. A randomly selected surface of each specimen was standardized by wet grinding with silicon carbide abrasive papers (grit P320 to P1200) on a tabletop grinding and polishing machine (Ecomet 6, Buehler, Lake Bluff, USA). Afterward, the specimens were cleaned in an ultrasonic bath with distilled water for 8 min and subsequently stored in distilled water at 37 °C for 24 hours (Chen et al. 2024, Rohym et al. 2023, Liebermann et al. 2017, Liebermann et al. 2019).

**Table 1:** Brand names, manufacturers, batch numbers and chemical composition of materials used

**Brand/Manufacturer**	**Abbreviation**	**Batch number**	**Chemical Composition**
Clearfil Majesty ES-2 Universal Kuraray Noritake Dental Inc, Okayama, Japan	CLA	430020	Silanated barium glass filler, pre-polymerized organic filler (0.0–40.0%), silanated colloidal silica, Bisphenol A diglycidylmethacrylate (5.0–15.0%), Hydrophobic aromatic dimethacrylate, Hydrophobic aliphatic dimethacrylate, dl-Camphorquinone, initiators, pigments
Ecosite One DMG Chemisch-Pharmazeutische Fabrik GmbH Hamburg, Germany	ECO	306409	Dental glass, EBPADMA (Ethoxylated bisphenol A dimethacrylate), SiO₂ (Silicon dioxide), neopentyl glycol propoxylate diacrylate (NPGDA), UDMA (Urethane dimethacrylate), additives, inorganic fillers: approximately 65 vol%, particle size 0.02–0.7 µm
Transcend Universal Composite Ultradent Products, Inc., South Jordan, UT, USA	TRA	BH5GZ	Dimethacrylate resin 1–21%, methacrylate resin 1–21%, silica 60–90%, silane treated silica

### Staining protocol

Each composite group was randomly assigned to one of four staining media (n = 10 per medium): **a**) Artificial saliva, **b**) Coffee, **c**) Red wine, **d**) Matcha tea, with their compositions described as follows. Artificial saliva was formulated according to established protocols and contained: 22.1 mmol/L bicarbonate, 16.1 mmol/L potassium, 14.1 mmol/L sodium hydrogen, 2.6 mmol/L phosphate, 0.8 mmol/L boric acid, 0.7 mmol/L calcium, 0.4 mmol/L thiocyanate, 0.2 mmol/L magnesium, pH: 7.4–7.8 (MANFRO). The coffee solution was prepared by brewing a Dallmayr Capsa Prodomo capsule (Alois Dallmayr Kaffee oHG, Munich, Germany) using a 19-bar capsule machine. The matcha tea solution was prepared by mixing 2 g of organic matcha powder (Health Bar GmbH, Berlin, Germany) with 100 mL of 80 °C hot water. The red wine used was a commercial Doppio Passo (Botter Casa Vinicola S.P.A., 13% vol. alcohol). All solutions were pre-warmed to 37 °C before the specimens were stored in these solutions at 37 °C in an incubator (Schülke Cultura, Norderstedt, Germany). The solutions were changed daily by the same operator (MJ) to prevent mold formation.

### Color measurements

After the staining procedure, color measurements were performed according to the eLAB system protocol (Hein et al. 2021). A custom-made acrylic plate (Palavit L, Kulzer, Hanau, Germany) was integrated into a phantom head (Frasaco GmbH, Tettnang, Germany) to ensure consistent positioning. A standardized white balance card (Emulation, Freiburg im Breisgau, Germany) was attached to the sample interface for color calibration. The imaging system consisted of a digital single-lens reflex camera (Canon EOS 6D Mark II) with a macro twin flash MT-24EX and a 100 mm f/2.8 macro lens (Canon Inc., Tokyo, Japan). The optimized camera settings were: Aperture: f/22, Exposure time: 1/125 s, ISO: 100 (HEIN). A cross-polarization filter (polar_eyes, Emulation, Freiburg im Breisgau, Germany) was used to reduce glare. All photographic components and camera parameters were standardized in accordance with the eLAB protocol. The use of a calibrated white balance card ensured consistent color rendering across all time points. No additional device calibration was necessary, as all equipment operated within factory-specified tolerances and standard measurement conditions.

Color measurements were conducted at five defined time points:

T0 (baseline), T1 (after 7 days), T2 (after 14 days), T3 (after 21 days), T4 (after 28 days).

The measured CIELAB color values (L, a, b*) were converted to LCH values (L, C, H***), and color differences were calculated according to the CIEDE2000 formula (ΔE₀₀):

$$\Delta {E}_{00}= \sqrt{\left\{ {\left(\frac{{\Delta L}{\prime}}{{k}_{L}{S}_{L}}\right)}^{2}{+\left(\frac{{\Delta C}{\prime}}{{k}_{c}{S}_{c}}\right)}^{2}+{\left(\frac{{\Delta H}{\prime}}{{k}_{H}{S}_{H}}\right)}^{2}+{R}_{T}\frac{{\Delta C}{\prime}}{{k}_{c}{S}_{c}}\frac{{\Delta H}{\prime}}{{k}_{H}{S}_{H}} \right\}}$$

where SL, SC, SH are scaling factors to adjust the perception sensitivity for brightness, chroma, and hue differences. kL, kC, kH are parameters to account for viewing conditions. RT is a rotation parameter to correct perceptual distortions, especially in the blue spectrum.

### Surface analysis

3D surface roughness was measured using a 3D laser scanning microscope (VK-X3050, Keyence Corporation, Osaka, Japan). Three random measurements per specimen were performed to calculate the mean values for the roughness parameters Sa and Sz.

### Data analysis

All statistical analyses were performed using RStudio (R Version 4.4.3). A linear mixed model (LMM) was applied to analyze the effects of composite type, staining solution, and time points on color change (ΔE₀₀) and surface roughness (Sa, Sz). Post-hoc tests were conducted using Tukey’s HSD test. Assumptions of normality and homogeneity of variances were checked. The significance level was set at α = 0.05.

## Results

### Color analysis

**Fig. 2:**
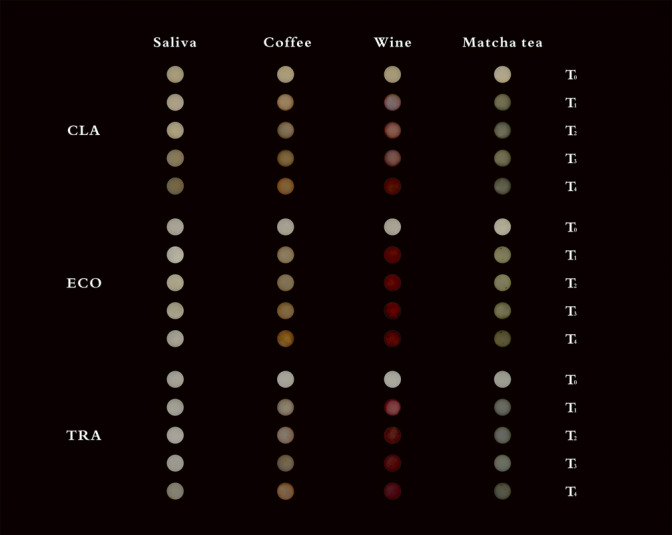
Representative photographs of test specimens from each group after immersion in staining solutions, illustrating visual differences in discoloration. Abbreviations: ECO, Ecosite One; TRA, Transcend Universal Composite; CLA, Clearfil Majesty ES-2; Immersion Times: T_0_: baseline; T_1_: after 7 days; T_2_: after 14 days; T_3_: after 21 days; T_4_: after 28 days

**Fig. 3:**
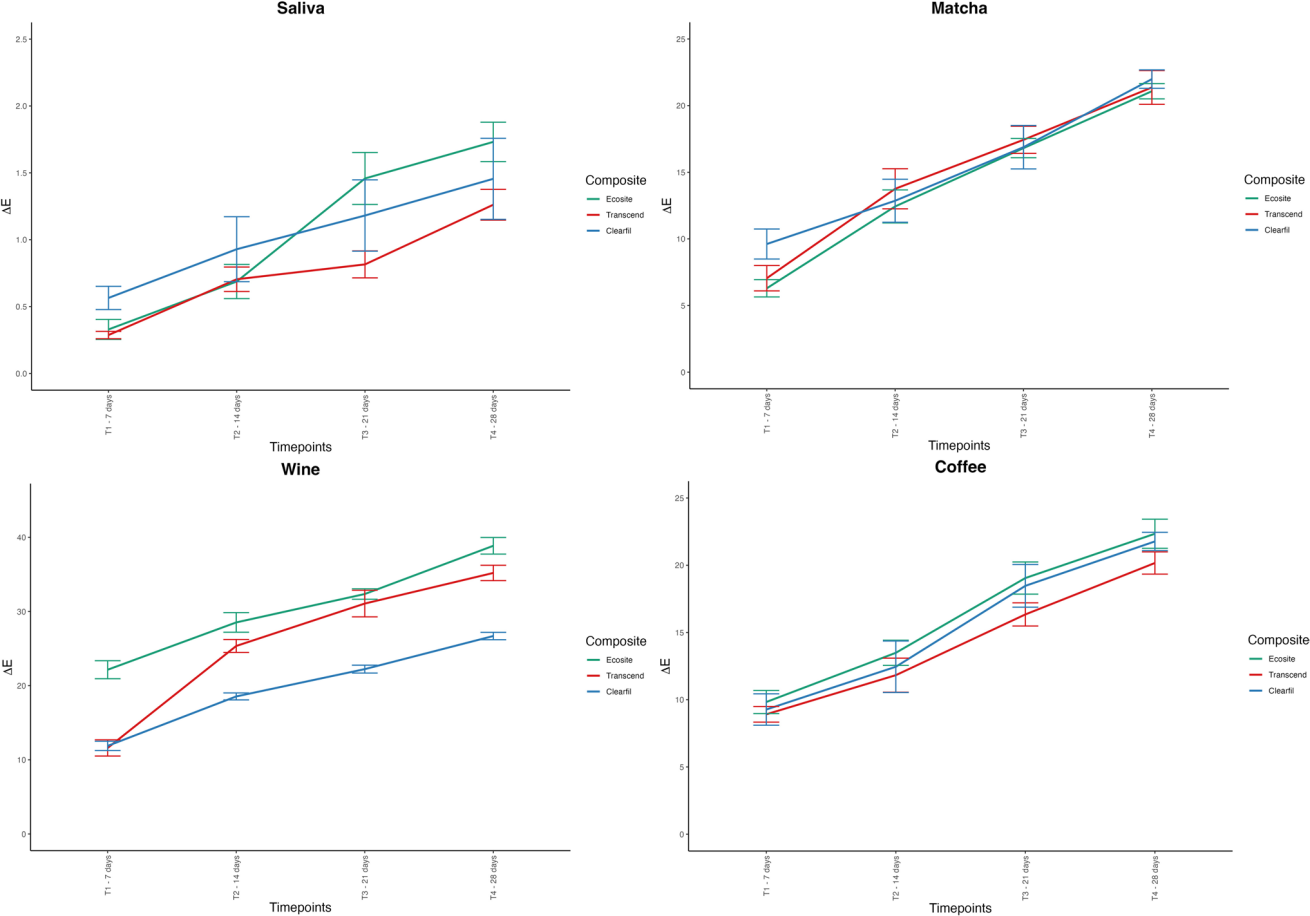
Line graph showing the mean ΔE₀₀ values and standard deviations of test specimens at different time intervals after immersion in staining solutions

The LMM analysis showed significant effects for composite type (F(2, 68.1), η^2^ = 0.558, p < 0.001), staining solution (F(3, 3145.6), η^2^ = 0.989, p < 0.001), and time points (F(3, 2570.6), η^2^ = 0.960, p < 0.001). Significant interactions were also observed for the combination of composite type and staining solution (F(6, 80.4), η^2^ = 0.817, p < 0.001), and composite type, staining solution, and time points (F(18, 16.0), η^2^ = 0.470, p < 0.001). Figure 2 shows the samples at different time points (T0–T4) and visualizes the differences in discoloration of the single-shade universal composites due to the various staining solutions. The highest ΔE₀₀ value was observed for red wine at T4 (ECO: 38.9 ± 1.56), followed by coffee (ECO: 22.3 ± 1.51) and matcha tea (CLA: 22.0 ± 0.97). The least color change was measured in artificial saliva at T1 (TRA: 0.29 ± 0.04). Post-hoc analysis using Tukey’s HSD test confirmed significant differences between the composites, time points, and staining solutions (p < 0.001). Coffee and matcha tea showed comparable discoloration potential (Table 2, Fig. 3).

**Table 2:** Mean ΔE00 values ± standard deviations of composites after 7, 14, 21 and 28 days of immersion in solutions

	ECO	TRA	CLA
**ΔE00 7d** p < 0.05	p < 0.05		
Saliva	0.33 ± 0.1	0.29 ± 0.04	0.56 ± 0.12
Matcha	6.29 ± 0.91	7.05 ± 1.33	9.62 ± 1.57
Coffee	9.83 ± 1.2	8.92 ± 0.81	9.27 ± 1.63
Wine	22.2 ± 1.7	11.6 ± 1.53^a^	11.9 ± 0.88
**ΔE00 14d** p < 0.05			
Saliva	0.69 ± 0.18**	0.7 ± 0.13**	0.93 ± 0.34**
Matcha	12.4 ± 1.73†	13.8 ± 2.1†	12.9 ± 2.26†
Coffee	13.5 ± 1.31†	11.8 ± 1.77†	12.4 ± 2.68†
Wine	28.5 ± 1.85**	25.4 ± 1.22**	18.6 ± 0.66**
**ΔE00 21d** p < 0.05			
Saliva	1.46 ± 0.27**	0.82 ± 0.14**	1.18 ± 0.37**
Matcha	16.8 ± 1.01†	17.4 ± 1.42†	16.9 ± 2.28†
Coffee	19.0 ± 1.67†	16.3 ± 1.21†	18.5 ± 2.22†
Wine	32.4 ± 0.99**	31.1 ± 2.51**	22.2 ± 0.75**
**ΔE00 28d** p < 0.05			
Saliva	1.73 ± 0.21**	1.26 ± 0.16**	1.46 ± 0.42**
Matcha	21.1 ± 0.8†	21.4 ± 1.77†	22.0 ± 0.97†
Coffee	22.3 ± 1.51†	20.2 ± 1.15†	21.8 ± 0.95†
Wine	38.9 ± 1.56**	35.2 ± 1.45**	26.7 ± 0.7**

Significant differences between Composites (p < 0.05), Timepoints and Media (p < 0.05), except for Matcha vs. Coffee (not significant, marked as `†`)

Abbreviations: ECO, Ecosite One; TRA, Transcend; CLA, Clearfil Majesty ES-2

### Surface roughness

**Fig. 4:**
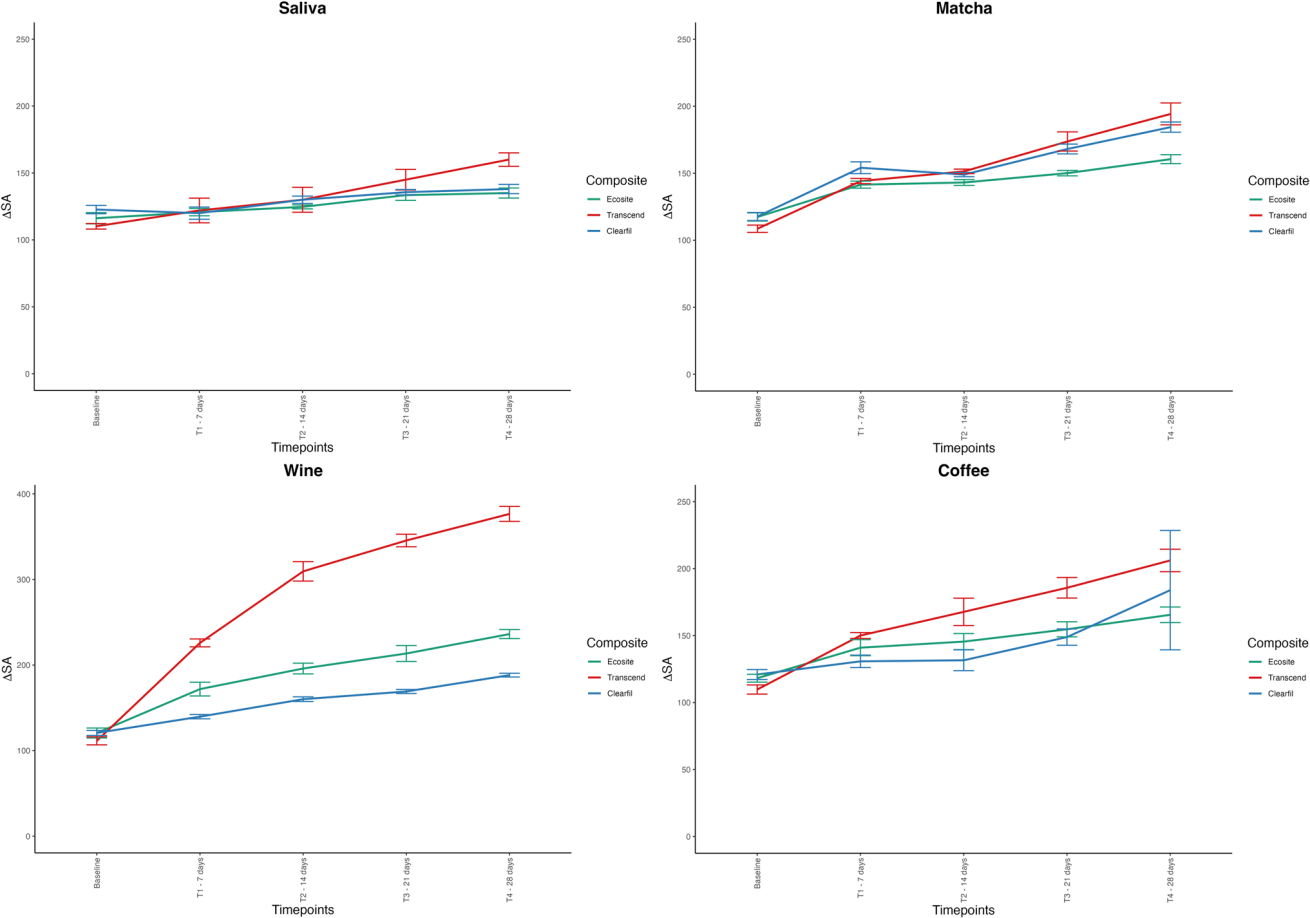
Line graph illustrating changes in surface roughness parameter (ΔSa) for all test groups at varying immersion times in staining solutions. Abbreviations: Sa: arithmetical mean height

Significant effects for composite type (F(2, 496.7), η^2^ = 0.902, p < 0.001), staining solution (F(3, 1048.6), η^2^ = 0.967, p < 0.001), and measurement time points (F(4, 942.2), η^2^ = 0.897, p < 0.001) were found. A significant interaction between the factors was also observed (F(24, 30.2), η^2^ = 0.627, p < 0.001). Figure 4 illustrates the progression of average surface roughness (Sa) defined as the arithmetical mean height over the entire staining period (T0–T4). A steep increase in Sa was observed in TRA and ECO specimens exposed to red wine, indicating pronounced surface degradation under acidic conditions. CLA maintained relatively stable Sa values throughout, suggesting greater resistance to surface erosion. The highest Sa values were recorded for TRA in red wine at T4 (377 ± 12.2 nm), while the highest Sz values were observed for ECO in red wine at T4 (3.85 ± 0.21 µm) (Table 3). Post-hoc analysis showed significant differences in roughness between all groups (p < 0.001), except for coffee and matcha tea, which exhibited comparable roughness values.

**Table 3:** Mean surface roughness values (Sa = arithmetical mean height; Sz = maximum peak-to-valley height) ± standard deviations of composites after 7, 14, 21 and 28 days of immersion in staining solutions

	**Composite**	**T0**	**T1**	**T2**	**T3**	**T4**
**Sa (nm)**	p < 0,05					
Saliva*	CLA	120 ± 6.38*	123 ± 4.34*	130 ± 3.81*	136 ± 2.83*	138 ± 4.79*
	ECO	116 ± 5.86*	121 ± 3.84*	125 ± 2.06*	133 ± 5.51*	135 ± 5.25*
	TRA	110 ± 2.89*	122 ± 12.9*	130 ± 13*	145 ± 10.7*	160 ± 7.05*
Matcha†	CLA	118 ± 4.21	149 ± 2.24	154 ± 6.15	168 ± 5.11	184 ± 5.32
	ECO	117 ± 4.3	141 ± 3.66	143 ± 3.06	150 ± 2.82	161 ± 4.64
	TRA	109 ± 3.8	144 ± 2.73	151 ± 2.37	174 ± 9.98	194 ± 11.4
Coffee†	CLA	121 ± 5.14	131 ± 6.5	132 ± 10.8	149 ± 8.51	184 ± 6.23
	ECO	118 ± 4.13	141 ± 8.39	146 ± 8.39	155 ± 7.86	166 ± 8.14
	TRA	110 ± 4.77	150 ± 3.1	168 ± 14.3	186 ± 10.7	206 ± 11.8
Wine*	CLA	121 ± 4.07*	140 ± 3.53*	160 ± 3.8*	169 ± 3.25*	188 ± 3.05*
	ECO	121 ± 8.13*	172 ± 11.2*	196 ± 8.78*	213 ± 13.0*	236 ± 7.37*
	TRA	111 ± 6.55*	226 ± 6.42*	310 ± 15.9*	346 ± 10.3*	377 ± 12.2*
**Sz **${\varvec{\mu}}$** m**						
Saliva*	CLA	1.12 ± 0.14*	1.55 ± 0.15*	1.68 ± 0.07*	1.79 ± 0.08*	1.93 ± 0.06*
	ECO	1.48 ± 0.06*	1.81 ± 0.03*	1.94 ± 0.05*	2.13 ± 0,04*	2.42 ± 0.07*
	TRA	1.41 ± 0.03*	1.57 ± 0.12*	1.59 ± 0.14*	1.71 ± 0.17*	1.78 ± 0.29*
Matcha	CLA	1.12 ± 0.03	1.50 ± 0.10	1.88 ± 0.09	2.10 ± 0.08	2.63 ± 0.11
	ECO	1.49 ± 0.03	1.75 ± 0.08	1.79 ± 0.16	2.02 ± 0.08	2.12 ± 0.11
	TRA	1.44 ± 0.05	1.71 ± 0.08	1.81 ± 0.08	1.88 ± 0.10	2.12 ± 0.11
Coffee	CLA	1.12 ± 0.06	1.75 ± 0.06	2.59 ± 0.13	2.74 ± 0.13	3.29 ± 0.12
	ECO	1.48 ± 0.04	2.08 ± 0.16	2.11 ± 0.07	2.55 ± 0.13	2.79 ± 0.12
	TRA	1.44 ± 0.11	1.86 ± 0.11	2.11 ± 0.09	2.21 ± 0.11	3.05 ± 0.10
Wine*	CLA	1.14 ± 0.05*	2.05 ± 0.16*	2.36 ± 0.07*	2.55 ± 0.13*	2.79 ± 0.12*
	ECO	1.51 ± 0.10*	2.98 ± 0.16*	3.11 ± 0.07*	3.49 ± 0.12*	3.85 ± 0.21*
	TRA	1.41 ± 0.06*	2.45 ± 0.16*	2.55 ± 0.13*	2.65 ± 0.07*	3.57 ± 0.21*

Significant differences between Composites (p < 0.05), Timepoints and Media (p < 0.05), except for Matcha vs. Coffee (not significant, marked as `†`)

Abbreviations: ECO, Ecosite One; TRA, Transcend; CLA, Clearfil Majesty ES-2; Immersion Times: T0: baseline; T1: after 7 days; T2: after 14 days; T3: after 21 days; T4: after 28 days

## Discussion

Both null hypotheses were rejected: composite discoloration and surface roughness were significantly influenced by material composition and the staining medium. Red wine caused the most pronounced discoloration, followed by coffee and matcha tea, while artificial saliva had a negligible effect. Variations among the composites underscore the importance of resin formulation and filler morphology in determining color stability—findings consistent with prior studies showing that acidic and polyphenol-rich beverages promote pigment interaction with the resin matrix and filler particles (Catelan et al. 2011; Ardu et al. 2009; Elmalawany et al. 2023).

### Impact of staining media on color stability

All tested single-shade universal composites demonstrated minimal discoloration in artificial saliva. As a neutral, non-pigmented aqueous medium, artificial saliva closely resembles distilled water and served as a control (Bétrisey et al. 2018, Erturk-Avunduk et al. 2022). The slight color changes observed were consistent with previously described water absorption effects and hygroscopic expansion of the resin matrix (Elmalawany et al. 2023). These minimal changes confirm good baseline color stability in non-pigmented environments. In contrast, red wine caused pronounced discoloration across all composites, with visible inter-material differences. This can be attributed to wine’s low pH, high pigment and tannin content, and alcohol concentration—all of which facilitate pigment diffusion into the resin matrix (Catelan et al. 2011). Coffee, although less acidic, also caused noticeable discoloration. As previously reported, the chromogenic components of coffee—primarily low-molecular yellow dyes—progressively stain composites through both adsorption and absorption mechanisms (Chen et al. 2024).

Matcha, a finely ground Japanese green tea (Camellia sinensis), has gained global popularity as a lifestyle beverage and contains high concentrations of chlorophyll, catechins, and polyphenols. Chlorophyll, the primary pigment, exhibits strong chemical stability, which may contribute to surface discoloration (Kochman et al. 2020). Additionally, catechins, particularly epigallocatechin-3-gallate (EGCG), have been shown to interact with composite surfaces, facilitating pigment retention and contributing to staining (Najman et al. 2023). Matcha tea induced moderate discoloration, comparable to coffee but slightly less intense. Interestingly, ECO and CLA responded similarly to matcha, suggesting that staining susceptibility may not be solely governed by the resin matrix but also by other compositional variables.

Material-specific differences were evident. ECO exhibited the highest ΔE₀₀ values, likely due to its resin matrix containing EBPADMA, a monomer with ether-linked oxygen groups that enhance hydrophilicity and water uptake. This increased water sorption facilitates deeper pigment infiltration. Despite incorporating NC-1 technology—a proprietary approach aiming to enhance shade adaptation by optimizing the refractive index of resin and filler phases—ECO did not demonstrate improved resistance to extrinsic discoloration. Similarly, its chameleon effect, designed to promote optical blending with surrounding tooth structure, did not enhance chemical resilience against staining agents. Comparable findings were reported for hydrophilic, HEMA-containing self-adhesive resin cements, which showed marked discoloration and surface degradation when exposed to chromogenic agents (2025). These parallels underscore the role of matrix hydrophilicity in staining susceptibility across composite-based materials.

In contrast, TRA and CLA showed better color stability. TRA’s Resin Particle Match technology aligns refractive indices of resin and fillers to achieve better optical outcomes while promoting a denser microstructure that may restrict pigment ingress. CLA's inclusion of silanized barium borosilicate glass and Bis-GMA-based dimethacrylates likely reduces water absorption and limits dye diffusion, contributing to its superior color stability.

Notably, color stability appears to be influenced not only by the specific resin matrix but also by the broader design concept of the composite. Several studies report that single-shade composites undergo up to 50% more discoloration than multi-shade materials, especially after exposure to coffee (ΔE₀₀: 8.76–17.57 vs. 2.80–12.56) (Chen et al. 2024). Multi-shade composites typically contain higher filler loads and silanized particles, conferring greater resistance to water uptake. In contrast, single-shade composites rely on translucency for shade adaptation, which may inadvertently increase permeability to hydrophilic staining agents. Moreover, the discoloration often proves irreversible, even after bleaching treatment (Chen et al. 2024; Ersöz et al. 2022).

### Surface roughness and its relationship to discoloration

While surface roughness is widely considered to enhance pigment retention and bacterial adhesion (Aktu and Ulusoy 2024), the present study did not reveal a consistent correlation between increased roughness and the degree of discoloration. This contrasts with findings by Schoppmeier et al., who observed a positive correlation between surface roughness and discoloration in resin-based composites specifically self-adhesive resin cements (2025). The discrepancy may be attributed to differences in monomer composition and hydrophilicity, as SARC’s typically contain more hydrophilic components such as HEMA, which facilitate both water sorption and pigment uptake. In contrast, the single-shade universal composites examined in this study may be more influenced by matrix chemistry and filler characteristics than by surface morphology alone.

TRA exhibited the greatest surface roughness increase, potentially reflecting acid-induced matrix erosion and filler particle dislodgment. However, it did not exhibit the highest discoloration, further emphasizing the multifactorial nature of composite degradation. Acidic media like red wine are known to induce structural changes in dental materials, including surface softening and filler-matrix separation (Ardu et al. 2009).

ECO also showed substantial roughness increase, likely due to its high water-absorbing UDMA and EBPADMA-based matrix, which can swell in moist conditions and compromise surface integrity. In contrast, CLA exhibited minimal surface change, which may be attributed to its robust, highly silanized filler network that resists hydrolytic and acidic degradation.

Collectively, these findings emphasize that both surface roughness and resin chemistry contribute to the aesthetic longevity of single-shade composites. Importantly, surface roughness alone is not a reliable predictor of discoloration, particularly in materials with chemically unstable matrices.

### Clinical implications

Given their inherent translucency and optical blending technologies, single-shade universal composites offer clinical advantages in shade matching. However, their susceptibility to extrinsic discoloration—particularly from polyphenol-rich and acidic beverages—raises concerns about long-term aesthetics, especially in high-consumption patients. The observed discoloration and surface degradation underscore the need for careful composite selection based on individual dietary habits. For patients who regularly consume coffee, tea, or red wine, materials like CLA with better resistance profiles may be preferable. In addition, clinicians should consider implementing regular re-polishing or professional cleaning procedures as part of maintenance therapy to mitigate surface degradation and pigment retention. Furthermore, the finding that bleaching does not fully restore the original shade supports the importance of preemptive material selection rather than relying on post-discoloration interventions (Chen et al. 2024).

### Limitations and future directions

This in vitro study, while controlled, cannot fully replicate the complex dynamics of the oral environment, including salivary flow, biofilm formation, and mechanical forces during mastication. These factors could exacerbate or mitigate discoloration and surface degradation in clinical settings. The relatively short 28-day immersion period provides insight into early-stage staining but does not capture the long-term cumulative effects. Additionally, limited information on the filler dimensions and morphology of ECO and TRA constrains the interpretation of their degradation mechanisms. Future research should incorporate dynamic simulation models that include microbial, chemical, and mechanical challenges reflective of real-world oral conditions. Detailed characterization of filler-matrix interactions and long-term in vivo studies are necessary to validate these findings and guide material optimization for clinical practice.

## Conclusion

Within the limitations of this study, it was concluded that:

1. The discoloration susceptibility of single-shade universal composites was influenced by material composition and the staining medium.
2. All tested composites exhibited measurable color changes when exposed to red wine, coffee, and matcha tea, with material-dependent variations in discoloration behavior. Red wine induced the most pronounced color changes, whereas coffee and matcha tea resulted in comparable levels of discoloration.
3. In patients with frequent consumption of highly pigmented beverages, the use of single-shade composites in esthetically demanding restorations warrants careful consideration.
4. For patients with a high consumption of pigmented or acidic beverages, clinicians should critically assess the indication for single-shade universal composites, particularly in esthetically demanding regions.
5. Materials such as Clearfil Majesty ES-2 Universal (CLA), which showed superior color and surface stability, may be better suited for long-term esthetic restorations in such cases.

## Data Availability

Data is available upon request.

## Declarations

### Permission to reproduce material from other sources

No material from other sources requiring permission has been used in this manuscript.
